# The Role of Accessible Hematological Markers in Bullous Pemphigoid: A Systematic Review

**DOI:** 10.3390/ijms27010340

**Published:** 2025-12-28

**Authors:** Aleksandra Małolepsza, Katarzyna Juczyńska, Anna Woźniacka, Joanna Brzeszczyńska, Agnieszka Żebrowska

**Affiliations:** 1 Department of Dermatology and Venereology, Medical University of Lodz, 90-647 Lodz, Poland; 2 Department of General Biochemistry, University of Lodz, 90-236 Lodz, Poland

**Keywords:** bullous pemphigoid, neutrophil to lymphocyte ratio, eosinophil count

## Abstract

Bullous pemphigoid (BP) is the most common autoimmune subepidermal blistering disease. In recent decades, an increasing incidence of BP has been reported. The rationale for this study arises from the limited availability of advanced immunopathological and serological assays for assessing disease activity in bullous pemphigoid across many clinical centers. This systematic review evaluates evidence regarding hematological markers derived from complete blood count (CBC), such as eosinophil count and neutrophil-to-lymphocyte ratio (NLR), in BP patients. The Ovid MEDLINE and EMBASE databases were searched for English-language peer-reviewed papers published until 2 May 2025. Sixteen studies involving 1775 patients were included. Eosinophil count consistently correlated with disease severity, clinical phenotype, treatment response, and relapse risk, while NLR showed potential as a prognostic and therapeutic marker. Given their accessibility and cost-effectiveness, these parameters may have practical value in the routine clinical management of BP.

## 1. Introduction

Bullous pemphigoid (BP) is the most common autoimmune subepidermal blistering disease. It is characterized by the presence of circulating autoantibodies directed against two hemidesmosomal components of the basement membrane: BP180 and BP230 [1]. Clinically, BP typically presents with tense blisters on erythematous or normal-looking skin; however, non-bullous variants may also occur [2]. The disease affects elderly individuals, with the highest incidence in individuals over 80 years old [3]. In recent decades, an increasing incidence of BP has been reported in counties such as the United Kingdom, France and Germany [4]. Interestingly, this rise is particularly notable in atypical variants, including the urticarial, eczematous and pruritus-only forms.

The pathogenesis of BP is primarily associated with autoantibodies directed against BP180, particularly of the IgG1 and IgG4 subclasses. These initiate the disease process by binding to the hemidesmosomal antigen located in the dermal–epidermal junction, activating the complement cascade, leading to the recruitment of inflammatory cells to the basement membrane zone; this inflammatory response is exacerbated by the release of neutrophil elastase, which cleaves BP180. In addition, IgE autoantibodies against BP180EC and BP230 can bind to mast cells, triggering their degranulation. This process further amplifies inflammation and promotes eosinophil recruitment. Although neutrophils play a critical role in subepidermal blister formation, the inflammatory infiltrate in BP in humans is typically dominated by eosinophils, with relatively few neutrophils present [5].

According to the guidelines for the management of BP established by the European Academy of Dermatology and Venereology (EADV), diagnosis requires the identification of three key components: (1) suggestive clinical features; (2) positive direct immunofluorescence (DIF); and (3) the presence of serum IgG antibodies labelling the epidermal side of 1 M NaCl-separated normal human skin (salt-split skin, SSS) by indirect immunofluorescence microscopy (IIF) and/or reactivity with BP180 and/or BP230 by ELISA or IIF [6]. In patients with BP, disease severity and quality of life can be evaluated using various scales, such as the Bullous Pemphigoid Disease Area Index (BPDAI) and the Dermatology Life Quality Index (DLQI) [7,8].

A recent systematic review and meta-analysis suggests that serum levels of anti-BP180 autoantibody may serve as supportive biomarkers for assessing disease activity in BP patients [9]. There has also been increasing interest in identifying simple hematological markers derived from routine blood tests, such as neutrophil count, lymphocyte count, eosinophil count, neutrophil-to-lymphocyte ratio (NLR), platelet-to-lymphocyte ratio (PLR) and mean platelet volume (MPV). These parameters, obtained from a standard complete blood count (CBC), have already been investigated as potential markers of inflammation, disease activity and prognosis in various medical conditions, including dermatological disorders such as psoriasis and systemic lupus erythematosus [10,11].

Emerging evidence suggests that these hematological markers may also have a clinical impact in autoimmune blistering diseases, including bullous pemphigoid. As the global population ages and the incidence of BP continues to rise, the identification of easily accessible and cost-effective prognostic markers will be of significant clinical value. This systematic review evaluates current evidence on the role of accessible hematological markers in BP, with a particular focus on their utility in diagnosis, monitoring disease severity, predicting treatment response and elucidating the underlying immunopathology.

## 2. Materials and Methods

A systematic review was conducted according to the updated Preferred Reporting Items for Systematic Reviews (PRISMA) guidelines [12] (Supplementary Materials). This study was prospectively registered in the International Platform of Registered Systematic Review and Meta-Analysis Protocols (INPLASY) under the registration number INPLASY202580042. The Ovid MEDLINE and EMBASE databases were searched for English-language peer-reviewed papers published from database inception to 2 May 2025. The titles and abstracts were searched and screened by two authors (AM, KJ). A full Boolean search strategy was used. In Ovid MEDLINE this was as follows: (“Neutrophils” OR “Neutrophil count” OR “Lymphocytes” OR “Lymphocyte count” OR Platelet* OR “Platelet count” OR “Eosinophil count” OR Eosinophilia OR “neutrophil-to-lymphocyte ratio” OR NLR OR “platelet-to-lymphocyte ratio” OR PLR OR “red cell distribution width” OR “red blood cell count” OR “full blood count” OR “complete blood count” OR CBC):ti,abAND (“Bullous pemphigoid” OR Pemphigoid):ti,ab LIMITS: humans, English language. In EMBASE we used: (‘neutrophil’/exp OR ‘neutrophil count’ OR ‘lymphocyte’/exp OR ‘lymphocyte count’ OR platelet* OR ‘platelet count’ OR ‘eosinophil count’ OR eosinophilia OR ‘neutrophil to lymphocyte ratio’ OR nlr OR ‘platelet to lymphocyte ratio’ OR plr OR ‘red cell distribution width’ OR ‘red blood cell count’ OR ‘full blood count’ OR ‘complete blood count’ OR cbc):ab,ti AND (‘bullous pemphigoid’/exp OR ‘bullous pemphigoid’ OR pemphigoid):ab,ti AND [english]/lim AND [human]/lim.

**Figure 1:**
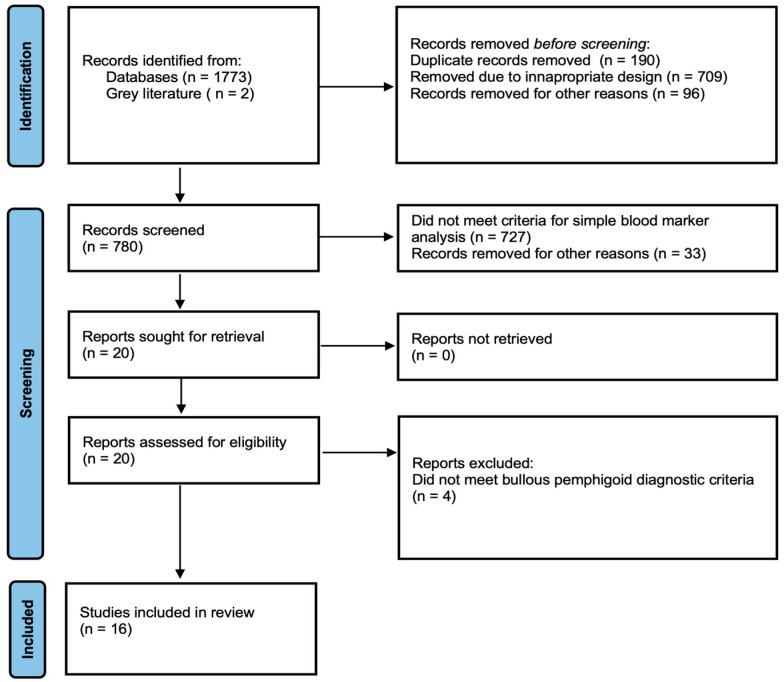
PRISMA flow diagram showing the systematic selection process of records.

Only studies published in English and conducted on human subjects were included in the systematic review. Non-clinical studies, reviews, case reports, unpublished data and conference reports were excluded. We assessed studies to ensure that the diagnosis of BP met the EADV guideline criteria, with particular emphasis on the presence of direct immunofluorescence (DIF). Studies in which the diagnosis was made based only on clinical and histopatological examination, or in which the method used to establish the diagnosis was not described at all, were not included (Figure 1).

One author (AM) extracted the data. Relevant data (authors, the date and type of the study, the number and demographics of the patients, the diagnostic method) were exported to a bespoke database. Microsoft Excel (Version 16.104, Microsoft, Redmond, WA, USA) was used to calculate the mean and SD of the age and male to female ratio.

All papers were assessed for methodological quality by two authors (AM, KJ) using the Newcastle–Ottawa Scale (NOS) [13]. The appropriate NOS tool was selected depending on whether the paper was a cohort study or a case-control study. The tool consists of eight items within three domains: selection of the study groups, comparability of the groups, and ascertainment of the outcome/exposure. The highest attainable score is nine stars. Studies assigned 7 to 9, 4 to 6, and 0 to 3 stars were categorized as high-quality, moderate-quality, and low-quality, respectively.

A meta-analysis could not be performed because the studies differed markedly in outcome definitions, severity scoring systems (including inconsistent use of BPDAI) and patient populations, which prevented the derivation of comparable effect estimates.

## 3. Results and Discussion

### 3.1. Results

The initial search yielded 778 publications. An additional 2 studies were identified through Google Scholar. After screening titles and abstracts, 20 studies were selected for full-text review (Figure 1). Following full-text assessment, four studies were excluded, resulting in a total of 16 studies that met the eligibility criteria. Among these, four were prospective, and the remaining twelve were retrospective in design. In total, the included studies encompassed 1775 patients of whom 1002 (56.5%) were female and 773 (43.5%) were male. The mean age of the participants was 74.7 years (SD = 3.9) (Table 1).

The most frequently analyzed parameter was eosinophil count, assessed in all included studies. Other hematological markers commonly evaluated were: neutrophil count—4 studies; lymphocyte count—4 studies; platelet count—4 studies; monocyte count—2 studies; basophil count—1 study; NLR (neutrophil-to-lymphocyte ratio)—3 studies; PLR (platelet-to-lymphocyte ratio)—3 studies; NER (neutrophil-to-eosinophil ratio)—1 studies; MLR (monocyte-to-lymphocyte ratio)—1 study; ELR (eosinophil-to-lymphocyte ratio)—1 study; PNR (platelet-to-neutrophil ratio)—1 study; MPV (mean platelet volume)—4 studies (Table 2).

Five studies investigated the relationship between eosinophil count and disease severity [18,20,22,26,28]. Four of these reported a significant correlation between peripheral eosinophil count and disease severity as measured by the Bullous Pemphigoid Disease Area Index (BPDAI). Two studies explored the association between eosinophil count and clinical presentation [19,23]. One study examined eosinophil counts in patients with neurological diseases [27].

Eosinophil counts were evaluated in the context of treatment response and disease relapse across four studies [14,16,19,23]. Two studies analyzed eosinophil levels in cases of BP triggered by dipeptidyl peptidase-4 inhibitors (DPP-4i) [19,24]. Additionally, two studies assessed the correlation between peripheral and tissue eosinophil counts [17,20]. Further, eosinophil counts were examined in relation to immunological markers, including BP180-specific autoantibodies and interleukin-17 (IL-17), in two studies [18,21]. Regarding NLR, two studies evaluated its levels before and after treatment [14,18]. One study compared NLR values between patients with bullous pemphigoid (BP) and pemphigus disease [15].

Based on the Newcastle–Ottawa Scale assessment, six studies (37%) were rated as high quality, including three cohort studies and three case-control studies. A total of ten studies were considered moderate quality (63%), with scores ranging from 5 to 6, all consisting of cohort studies (Table 3 and Table 4).

### 3.2. Discussion

This is the first systematic review to analyse the role of hematological markers derived from the complete blood count in patients with bullous pemphigoid in the available literature. It summarizes the role of hematological markers in the setting of BP as diagnostic tools, indicators of disease severity, prognostic factors, or as correlates of specific disease clinical phenotypes. Previous systematic reviews have focused on complex biomarkers that are less routinely available. Compared with Geng et al., our review focuses on globally available and inexpensive biomarkers derived from a standard complete blood count, making them far more accessible in everyday clinical practice [30]. This provides a more practical and immediately applicable approach for clinicians managing bullous pemphigoid.

#### 3.2.1. Eosinophil Count and Eosinophil-to-Lymphocyte Ratio

The first reports of eosinophilia in BP originated from uncontrolled retrospective cohort studies, indicating its presence in approximately 50–70% of patients [31,32]. This observation aligns with the findings reported in studies included in this review [14,24].

Four of the included studies showed a significant correlation between peripheral eosinophil count and disease severity, as assessed by the BPDAI [18,20,22,26]. While the BPDAI scale is not routinely used in everyday clinical practice due to its complexity, eosinophil count is a widely accessible parameter and may serve as a practical surrogate marker for disease severity.

Interestingly, both Kridin and Garrido reported a higher frequency of acral involvement in BP patients with elevated eosinophil count [19,24]. Kridin also observed that these patients were older at the time of diagnosis, while individuals with normal eosinophil levels tended to be younger and more often presented with lesions affecting the mucous membranes and head and neck regions. Gambichler et al. report that BP patients with neurological disorders (BP + ND) tended to be older and demonstrated significantly higher eosinophil counts than a group without such comorbidities (BP–ND). This could suggest that eosinophil count might be associated with the presence of neurological disorders in patients with BP, or their future development and, as such, it could represent a potential marker of neurological involvement in this group.

Another promising finding is that eosinophil count and ELR may serve as a potential biomarkers for monitoring response to treatment with tetracyclines and for assessing remission status; at the end of treatment, patients in remission showed significantly reduced eosinophil counts and ELR compared to baseline levels [14,16]. Additionally, disease relapse was more frequently observed in patients who had elevated peripheral eosinophil counts at the time of diagnosis [19].

A drug-induced variant of bullous pemphigoid also exists, which can be triggered by dipeptidyl peptidase-4 inhibitors; this is particularly common among elderly diabetic patients [29]. Our present analysis indicates that patients with DPP-4i-associated BP were less likely to exhibit elevated peripheral eosinophil counts compared to those with idiopathic, which may reflect differences in the underlying immunopathogenesis [19,23].

Upon closer examination of the immunologic profile, patients with DPP-i-associated BP appear to demonstrate a shift in autoantibody specificity, with reactivity primarily targeting the mid-portion of BP180 rather than the immunodominant NC16A domain [33]. It was also demonstrated that DPP4 inhibitors, including vildagliptin, significantly downregulate key dermal–epidermal adhesion genes (LAMA3, LAMB3, LAMC2, DST, and COL17A1) in keratinocytes. These findings support a pathogenic model in which reduced expression of these structural components leads to basement membrane fragility and enables subepidermal blistering with minimal inflammation [34]. On the other hand, it has been shown that IL-6 expression can be induced by vildagliptin in keratinocytes leading to its accumulation in the skin [26]. This accumulation may further stimulate keratinocytes to produce additional IL-6, thereby amplifying inflammatory cascades in DPP-i-associated BP [35]. These findings explained skin lesion formation despite the fact that few eosinophils are present in histopathological DPP-i-associated BP skin biopsies.

In terms of clinical presentation, several studies have shown that disease severity in DPP-i-associated BP is comparable to that observed in idiopathic BP [36,37]. The first mentioned group of patients tend to present with more extensive erosions and blisters while exhibiting milder urticarial and erythematous lesions [34]. A point of particular significance is that reported median latencies between the initiation of DPP-4 inhibitor therapy and the onset of BP vary widely across studies, ranging from 6 to 26 months [23,38,39]. This broad interval indicates that DPP-4 inhibitors may still be considered a potential precipitating factor even when exposure has exceeded two years prior to clinical presentation.

DPP inhibitors have been associated with an increased risk of bullous pemphigoid, particularly its non-inflammatory variants, which result from distinct immunopathogenic mechanisms; moreover, because the latency between treatment initiation and the onset of cutaneous lesions can be prolonged, clinical vigilance is warranted in patients receiving these drugs.

#### 3.2.2. Neutrophil-to-Lymphocyte Ratio

One study demonstrated significantly higher NLR values in patients with BP compared to healthy controls. However, this study was not included in the review due to the diagnosis being based solely on clinical and histopathological examination findings, without immunopathological confirmation [40].

Valuable insights into the role of NLR in assessing BP were provided by Sun et al. Their study of 36 untreated BP patients found elevated NLR levels to be significantly associated with higher BPDAI Erosion/Blister Score and BPDAI Total Score. Furthermore, NLR was evaluated at five sequential time points during treatment, and it remained positively correlated with both BPDAI Erosion/Blister Score and Damage Score throughout the course of therapy. These findings suggest that NLR may be a useful marker for monitoring treatment response and predicting disease prognosis in BP. Similar observations have been made in other inflammatory skin conditions, such as psoriasis [41,42]. Additionally, a study assessing NLR after treatment initiation in pemphigus vulgaris (PV) found a significant reduction in NLR values by the third month of therapy compared to baseline [43]. In that study, NLR also showed a significant positive correlation with PDAI scores at months 3 and 6. However, data on NLR in autoimmune blistering diseases, especially bullous pemphigoid, remain limited.

In contrast, Avcı et al. did not find any significant difference in NLR values before and after treatment in a cohort of 50 BP patients treated with tetracycline-based antibiotics [14]. A comparative study by Rai et al. found that NLR was significantly higher in BP than in pemphigus disease, suggesting a stronger neutrophil-driven inflammatory response in BP, which aligns with the known involvement of both eosinophils and neutrophils in its subepidermal blistering pathogenesis [15]. In the same study, an NLR cut-off value ≥ 7 had high specificity (90.6%) but low sensitivity (22.7%) for distinguishing BP from pemphigus disease.. This indicates that while a high NLR may support a diagnosis of BP, it is not reliable as a standalone diagnostic marker.

A particularly interesting study conducted in a Thai population found both anemia and elevated NLR to be associated with 3-year mortality in BP patients [44]. However, this study was excluded from our review due to the lack of standardized immunopathological criteria used for confirming the BP diagnosis.

#### 3.2.3. Other Markers

Other hematological parameters have been reported less frequently in the literature and are often limited to individual studies. Nonetheless, certain clinically relevant findings warrant attention.

One study reported that peripheral blood monocyte counts were significantly lower in patients who remained in remission after tetracycline treatment compared to those who experienced relapse [14]. This reduction suggests that, alongside eosinophil count, monocyte levels may serve as a potential marker of disease activity in BP and could help predict the risk of relapse.

A lower NER in patients with bullous pemphigoid compared to those with pemphigus disease could reflect fundamental differences in the pathophysiology of these two autoimmune blistering diseases [14]. In BP, eosinophils play a central pathogenic role and are frequently found in both skin lesions and peripheral blood. In contrast, PV typically exhibits a predominance of neutrophils and lymphocytes, with less pronounced eosinophilia.

Mean platelet volume (MPV), a parameter that reflects platelet size and is commonly used as an indicator of platelet activation and systemic inflammation, has also been investigated in BP [45]. In a study by Sahin et al., MPV values significantly decreased during remission in BP [16]; in contrast, Rifaioglu et al. found no significant difference in MPV levels before and after treatment, although they noted higher MPV values in BP patients compared to healthy controls [46]. However, this study was excluded from the current systematic review due to reliance on clinical and histopathological criteria alone for diagnosis. Additionally, Sun et al. did not identify any correlation between MPV and disease severity or treatment response [18]. No significant associations between platelet-to-lymphocyte ratio or monocyte-to-lymphocyte ratio and bullous pemphigoid were identified in the reviewed studies [14,15].

## 4. Conclusions

Numerous simple and readily accessible parameters can be derived from the complete blood count or calculated from the ratios between its components. Among these, eosinophil count appears to be the most promising marker in BP. Elevated eosinophil levels are associated with disease severity and characteristic clinical manifestations, and may also be useful in monitoring disease progression and predicting the risk of relapse.

In contrast, while findings on the NLR are not entirely consistent, NLR remains an easily obtainable parameter that may serve as a supplementary marker for assessing treatment response. Nevertheless, further prospective studies involving larger patient cohorts are required to validate its clinical utility and to clarify its role in the management of BP.

While this review provides a new insight into the role of hematological markers in the diagnosis of BP, it should be noted that the interpretation of hematologic markers must consider potential confounders—age, comorbidities, concomitant infections, and treatments, especially systemic corticosteroids. The value of these parameters lies not in being disease-specific markers, but in providing accessible, low-cost indicators that may support clinical assessment when interpreted in context. Corticosteroids are known to influence hematological parameters, and while several included studies reported that patients were treatment-naive at the time of blood sampling, this was not consistently documented across all studies; therefore, variability in treatment status may have affected some of the reported hematological values. The majority of included studies were retrospective in design. As a result, many of the proposed hematological markers could not be evaluated longitudinally, particularly in relation to treatment initiation or disease relapse.

In this study, we present the overall trends in biomarker levels among patients with bullous pemphigoid, focusing on whether specific biomarkers are consistently elevated or reduced rather than on exact quantitative values. Nevertheless, further well-designed studies are required to validate these observations and better define their clinical and therapeutic relevance.

## Figures and Tables

**Table 1:** Descriptive data of the included studies.

Study	Study Design	Sample Size (BP)	Sex (M:F)	Mean Age (Years)	Diagnostic Methods
Avcı et al., 2024 (Turkey) [14]	Retrospective	50	17:33	70.9	Clinical presentation, histopathology and DIF.
Rai et al., 2023 (India) [15]	Retrospective	66	-	64	Histopathology and DIF.
Sahin et al., 2023 (Turkey) [16]	Retrospective	46	18:28	76.67	Histopathology and DIF.
Baum et al., 2023 (Israel) [17]	Retrospective	137	60:77	75.23	Clinical presentation, histopathology and DIF.
Sun et al., 2023 (China) [18]	Prospective	36	24:12	74.6	First criterion and at least two of the last three criteria: (1) Clinical presentation, (2) DIF, (3) ssIIF, (4) ELISA for BP180 NC16A.
Garrido et al., 2022 (Portugal) [19]	Retrospective	233	123:110	79.3	Clinical presentation, histopathology, DIF and ELISA.
Karaali et al., 2021 (Turkey) [20]	Retrospective	59	21:38	75.9	Clinical presentation, DIF, IIF, salt-split and ELISA.
Delli et al., 2020 (Greece) [21]	Prospective	30	20:10	77.9	Clinical presentation, histopathology, DIF and ELISA.
Farnaghi et al., 2020 (Iran) [22]	Prospective	27	13:14	71.77	Clinical presentation, histopathology and DIF.
Kridin, 2020 (Israel) [23]	Retrospective	397	174:223	78	Clinical presentation, histopathology, at least one of the following: (1) DIF, (2) IIF, (3) ELISA.
Kridin, 2018 (Israel) [24]	Prospective	225	111:114	77.2	Clinical presentation, histopathology, at least one of the following: (1) DIF, (2) IIF, (3) ELISA
Park et al., 2018 (Korea) [25]	Retrospective	60	34:26	74.7	Clinical presentation, histopathology, DIF, IIF and salt-split.
van Beek et al., 2016 (Germany) [26]	Retrospective	153	70:83	75.76	Clinical presentation, DIF or salt-split and ELISA.
Gambichler et al., 2015 (Germany) [27]	Retrospective	161	43:118	81.5	Clinical presentation, histopathology and DIF.
Messingham et al., 2014 (USA) [28]	Retrospective	48	25:23	78.2	Clinical presentation, histopathology and DIF.
Lee et al., 2012 (USA, Korea) [29]	Retrospective	47	20:27	71.5	Clinical presentation, histopathology, DIF, IIF and salt-split.

**Table 2:** Assessed markers in the included studies.

Study	Assessed Markers
Avcı et al., 2024 [14] (Turkey)	Neutrophil count
	Lymphocyte count
	Eosinophil count
	Platelet count
	Monocyte count
	NLR
	ELR
	MLR
	PLR
	MPV
Rai et al., 2023 [15] (India)	Neutrophil count
	Lymphocyte count
	Eosinophil count
	Platelet count
	NLR
	NER
	PLR
Sahin et al., 2023 [16] (Turkey)	Eosinophil count
	Basophil count
	Platelet count
	MPV
Baum et al., 2023 [17] (Israel)	Eosinophil count
Sun et al., 2022 [18] (China)	Neutrophil count
	Lymphocyte count
	Eosinophil count
	Platelet count
	NLR
	PLR
	PNR
	MPV
Garrido et al., 2022 [19] (Portugal)	Eosinophil count
Karaali et al., 2021 [20] (Turkey)	Eosinophil count
Delli et al., 2020 [21] (Greece)	Eosinophil count
Farnaghi et al., 2020 [22] (Iran)	Eosinophil count
Kridin, 2020 [23] (Israel)	Eosinophil count
Kridin, 2018 [24] (Israel)	Eosinophil count
Park et al., 2018 [25] (Korea)	Neutrophil count
	Lymphocyte count
	Eosinophil count
	Monocyte count
van Beek et al., 2016 [26] (Germany)	Eosinophil count
Gambichler et al., 2015 [27] (Germany)	Eosinophil count
Messingham et al., 2014 [28] (USA)	Eosinophil count
Lee et al., 2012 [29] (USA, Korea)	Eosinophil count

**Table 3:** Newcastle–Ottawa Scale assessment of the quality of the cohort studies. Dashes signify that there was no documented parameter related to the column heading in the paper reviewed.

Study	Representativeness of Exposed Cohort	Selection of Non-Exposed Cohort	Ascertainment of Exposure	Outcome of Interest Was Not Present at Start of Study	Comparability of Cohorts	Assessment of Outcome	Appropriate Follow-Up Length	Adequacy of Follow-Up of Cohorts	Total Score (Out of 9)
Avcı et al. [14]	★	-	★	★	★★	★	★	★	8
Sahin et al. [15]	★	-	★	★	★	★	★	-	5
Baum et al. [17]	★	-	★	★	★★	★	★	★	8
Sun et al. [18]	★	-	★	★	★	★	★	-	6
Garrido et al. [19]	★	-	★	★	★	★	★	-	6
Karaali et al. [20]	★	-	★	-	★★	★	★	-	6
Delli et al. [21]	★	-	★	★	★★	★	-	-	6
Farnagi et al. [22]	★	-	★	-	★★	★	-	-	5
Kridin et al. [23] (2020)	★	★	★	★	★	★	-	-	6
Park et al. [25]	★	-	★	★	★	★	-	-	5
Gambichler et al. [27]	★	★	★	★	★	★	-	-	6
Messingham et al. [28]	★	★	★	★	★	★	-	-	6
Lee et al. [29]	★	★	★	★	★	★	★	★	8

★ One star was awarded if age was controlled for in studies in the comparability of cohorts on the basis of the design or analysis section. ★★ A further star was awarded if coexisting medical conditions were reported in the study.

**Table 4:** Newcastle–Ottawa Scale assessment of the quality of the case-control studies. Dashes signify that there was no documented parameter related to the column heading in the paper reviewed.

Study	Is the Case Definition Adequate?	Representativeness of the Cases	Selection of Controls	Definition of Controls	Comparability of Cases and Controls	Ascertainment of Exposure	Ascertainment for Cases and Controls	Non-Response Rate	Total Score (Out of 9)
Rai et al. [15]	★	★	★	★	★	★	★	-	7
Kridin [24] (2018)	★	★	★	★	★★	★	★	★	9
van Beek et al. [26]	★	★	★	★	★	★	★	-	7

★ One star was awarded if age was controlled for in studies in the comparability of cohorts on the basis of the design or analysis section. ★★ A further star was awarded if coexisting medical conditions were reported in the study.

## Data Availability

No new data were created or analyzed in this study. Data sharing is not applicable to this article.

## Supplementary Materials

The supporting information can be downloaded at: https://www.mdpi.com/article/10.3390/ijms27010340/s1.
